# Applications of a Novel Tunable Piezoelectric Vibration Energy Harvester

**DOI:** 10.3390/mi14091782

**Published:** 2023-09-17

**Authors:** Sreekumari Raghavan, Rishi Gupta, Loveleen Sharma

**Affiliations:** Department of Civil Engineering, Engineering Computer Science (ECS) #314, University of Victoria, Victoria, BC V8W 2Y2, Canada; guptar@uvic.ca (R.G.); loveleensharma@uvic.ca (L.S.)

**Keywords:** vibrations, energy harvesting, piezoelectric material, applications of piezoelectric vibration energy harvester (PVEH), ionic polymer–metal composite (IPMC)

## Abstract

Conversion of ambient energy to usable electrical energy is attracting attention from researchers since providing a maintenance-free power source for the sensors is critical in any IoT (Internet of Things)-based system and in SHM (structural health monitoring). Continuous health monitoring of structures is advantageous since the damage can be identified at inception and the necessary action taken. Sensor technology has advanced significantly, and MEMS (microelectromechanical systems)-based low-power sensors are available for incorporating into large structures. Relevant signal conditioning and transmission modules have also evolved, making them power-efficient and miniaturized. Various micro wireless sensor nodes (WSN) have also been developed in recent years that require very little power. This paper describes the applications of a novel tunable piezoelectric vibration energy harvester (PVEH) for providing autonomous power to low-power MEMS sensors for use in IoT and remote SHM. The novel device uses piezoelectric material and an ionic polymer–metal composite (IPMC) and enables electrical tuning of the resonant frequency using a small portion of the power generated.

## 1. Introduction

In recent years, there has been a radical change in the concept of monitoring the health of systems and structures. The concept is to deploy a very large number of sensors connected to the internet and have them communicate with each other. The Internet of Things (IoT) has revolutionized the process by which systems across the world are monitored and controlled. The sensors in microscale microelectromechanical systems (MEMS) utilize very little power, making the above concept feasible. However, the main issue associated with the scheme is the mode of powering the sensors. Batteries with wired connections to a central power supply are not a feasible option in all cases. Further sensors will need to be deployed under inaccessible and hostile conditions on host structures. In order to overcome these issues, generating useful power from ambient conditions and its application in diverse areas have been explored by many researchers. Some of the research reported is detailed below.

Alla et al. [1] discussed piezoelectric vibration energy harvesting for powering the sensors embedded for monitoring tire pressure in automobiles. Hosseinkhani et al. [2] provided a comprehensive review of using sound- and piezo-based vibration energy harvesting in railways by providing different methods and their advantages and drawbacks. Esmaeeli et al. [3] reported their study on a rainbow-shaped piezoelectric vibration energy harvester mounted on the tire to power the sensors deployed for the structural health monitoring of tires. Goel et al. [4] came up with the application of piezoelectric vibration energy harvesters in space vehicles, where the main source of power is large and heavy solar panels. Their research recommends the use of vibration energy harvesters, as they are lighter and more cost-effective for space applications. Beeby et al. [5] reported the implementation of piezoelectric vibration energy harvesters in rail and industrial applications. The miniaturization of piezoelectric harvesters was realized using a screen-printing method for printing the device entirely. Khan et al. [6] worked on applications for converting bridge vibrations into useful power by adapting piezoelectric energy harvester configurations. Bridge vibrations are in the low frequency range with low acceleration levels. Ali et al. [7] covered in their review the recent advances in piezoelectric vibration energy harvesters and the implantable medical devices that can be powered by them. Implantable medical devices are being used to manage critical human health conditions. The kinetic energy generated by muscle relaxation, blood flow, cardiac movements, lung movements, etc., can be harvested using vibration energy harvesters. Hwang et al. [8] described flexible thin-film piezoelectric energy harvesters and nanosensors for biomedical applications. Inorganic, biocompatible piezoelectric materials are being used for implantable devices. Panda et al. [9] described the various applications of piezoelectric vibration energy harvesters in biomedical applications such as cell stimulation, stimulation of the brain, and tissue engineering. The article also details piezoelectric biomaterials. Han et al. [10] demonstrated a 3D piezoelectric microsystem that can be used for sensing mechanical properties and energy conversion. The article also details the applications for realizing multi-functional sensors for robotic prosthetic interfaces. Mhetre et al. [11] reviewed various methods in the literature, mainly for piezoelectric energy harvesting, and discussed the need for improving the power output. This is even more relevant in the case of human energy conversion, which is needed for powering implantable devices. Zheng et al. [12] described the various structural designs of piezoelectric energy harvesters to harvest energy from biomechanical movements to power devices internally. It is emphasized that future work should be focused on flexible, stretchable, and biodegradable systems that are autonomously powered. Townsend et al. [13] worked on a piezoelectric vibration energy harvester to power the sensors on an aircraft. Aircraft systems generate localized vibrations at various frequencies. The work reported here is about generating considerable charges from different scenarios. Maruccio et al. [14] conducted a case study in which health monitoring of a cable-stayed bridge located in Italy was carried out using the harvested power from ambient vibrations. The vibration energy harvester that was deployed was made of arrays of electrospun piezoelectric nanofibers.

The low-power sensors developed in recent decades enable us to effectively integrate them into structures. Aspects to be considered are power requirements, the characteristics of the structures on which they have to be deployed, and the operating conditions. It can be seen that most of the machinery, structures, and moving systems undergo some sort of vibration during their life cycle. By tapping this vibration energy, an autonomous power source for powering the sensors on it can be realized.

In all the above applications, PVEH in cantilever mode is useful since the output at its resonance is reasonably high. However, the issue associated with this is that the PVEH generates high enough output only at its resonant frequency [15]. This has led to various designs of PVEH by many researchers to overcome this issue. But an active method with net power gain to broaden its frequency response is not reported in the literature.

Here, the authors have used a novel tunable piezoelectric vibration energy harvester, where a low-power Ionic Polymer Metal Composite (IPMC) actuator is used as a stopper, to power commercially available sensors. Details of the harvester are given in the earlier papers by the authors [16,17]. The authors also have a patent pending [18]. The highlights are detailed below:

- The resonant frequency of the harvester can be tuned electrically.
- IPMC, used as a stopper for tuning, consumes only about 25 to 50 µW for activation and much less to keep it in that state if required for a longer period.
- Part of the power generated by the harvester can be used for powering the IPMC stopper, which functions on a 1 to 4 V supply.
- The power generated by the harvester can be used to trickle charge a supercapacitor to store the generated power. This power can then be used to power sensors and data transmission circuits periodically and for short durations as required.
- In the application scenarios presented here, the IPMC was powered manually from power generated by the harvester. The sensor and transmission circuits were powered manually using the generated power from the harvester for the duration required for the transmission of data.
- A supercapacitor circuit was used to store the power generated in application 3. Once the supercapacitor is charged, every measurement brings down the supercapacitor voltage by about 0.2 V. The unit takes about 10 to 15 min of vibrations to make up for the power used. Charging the supercapacitor from 0 to 3 V took about three hours and twenty minutes.
- The same harvester was used for all the application scenarios presented.
- The device is amenable to fabrication in MEMS form. When this is undertaken, it can be integrated with a low-power transmitter, a low-power microcontroller, and low-power sensors as required for autonomous operation.

IPMC technology has rapidly evolved. The power required for activation is coming down, and higher blocking forces have been realized. Some of the relevant literature is given below. This indicates the possibility of improving on the present design of the harvester, which uses only commercially available components.

Punning et al. [19] reported that by appropriately choosing the input waveform, the power consumption of the IPMC can be significantly reduced. It is reported that a square wave modulated with asymmetric impulses can be applied to the IPMC for better performance and to reduce energy consumption. The frequency of the modulating impulses should be much lower than the response time of the IPMC in order to obtain the best response. Another feature is that the modulating impulses have a sharp rising edge and a sloping falling edge. It is also reported by Wang et al. [20] that the driving voltage of IPMC can be reduced by using a silver electrode, which has a low resistance and large ductility, avoiding possible electrolysis. The blocking force of the actuator is dependent on its thickness. Hence, a great deal of research has been carried out to improve the blocking force by introducing thickness-controlled ion exchange membranes. Performance is also enhanced by going for nano-dispersed metal electrodes instead of coated metal electrodes. Thicker membranes are realized by stacking pre-extruded Nafion films. IPMC films show remarkable displacement under relatively low voltage, using very low power, as reported by Shahinpoor et al. [21]. It has been established that by modifying the chemical composition of IPMC, the blocking force is greatly improved. Kim et al. [22] demonstrated that enhanced force by IPMC as an actuator can be realized by increasing the thickness of the film. Normally, a non-corrosive material is used as an electrode. In the study reported by Tamagawa et al. [23], silver, a corrosive metal, is used as an IPMC electrode. The reversible redox reaction of the silver electrode results in the material characteristics of the IPMC changing, which results in higher curvature and blocking force. Nemat Nasser et al. [24] have described how the IPMC materials are different from other electroactive polymers. The most unique aspect of IPMCs is that their actuation is based on the active motion of ions and solvent molecules within the membrane under applied voltage or force. The mechanism in IPMC is thus anisotropic or directional, whereas other materials exhibit passive and isotropic responses to stimuli. The performance of IPMCs as actuators depends on their surface morphology. The best electrodes for IPMC actuators will be those that have the largest available surface area and maximum conductivity. Paquette et al. [25] demonstrated that configurations with multilayers are also advantageous to achieve better actuation characteristics.

In our novel PVEH, commercially available components are used. In the applications presented also, commercially available devices are used.

## 2. Materials and Methods

**Figure 1:**
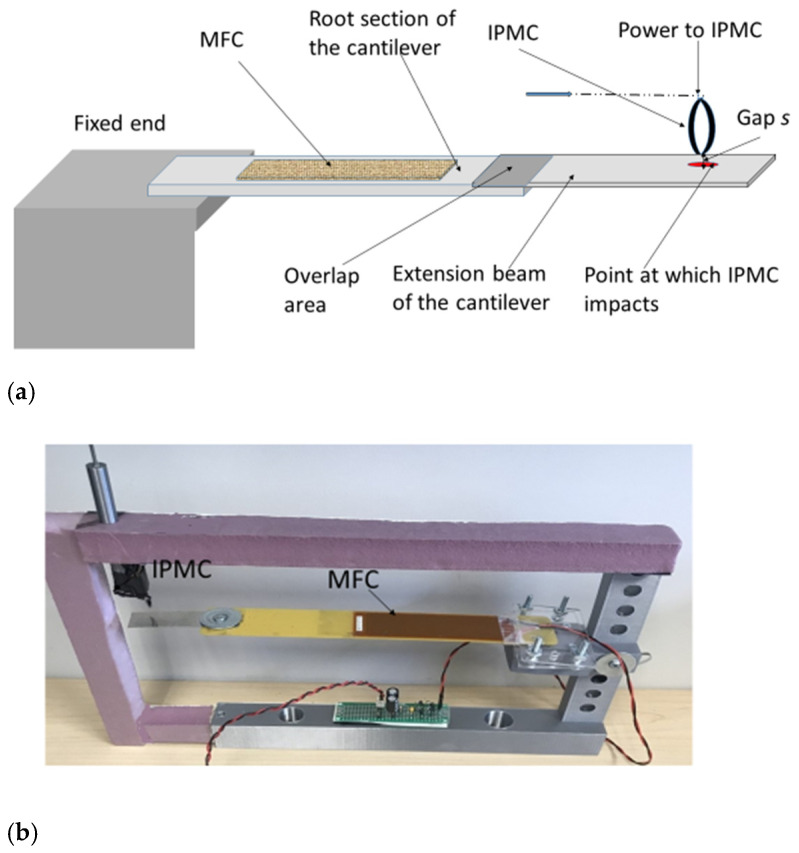
(**a**) Schematics and (**b**) Photograph of Novel tunable PVEH.

The novel device consists of a two-segment cantilever beam (Figure 1a,b) with a root section and an extension beam attached to it. The extension beam is thinner compared to the root section and is sensitive to even small impact loads from a stopper. An actuator arrangement made of two IPMC strips attached at the tips is used as a stopper to generate an impact force on the cantilever beam. IPMC, acting as a stopper, enables a broader frequency response due to its nonlinear response. The IPMC response can be changed by powering it from the same harvester, as it consumes very little power. This is akin to changing the properties of the stopper material. The piezoelectric material used is MFC (Macro Fiber Composite) from Smart Material Corporation [26], and IPMC is from Environmental Robots USA [27]. The device has a patent pending [18]. The novelty of the device, design details, and detailed experimental results are covered in earlier published papers by the authors [16,17].

The details of the PVEH used in the applications described in this paper are given below.

The root section is made of composite material (glass fiber FR4) of length 22 cm and width 3.3 cm. The extension beam is aluminum, with a length of 9.5 cm and a width of 2 cm. The extension beam is bonded to the root section using 3M465 film tape. The MFC used is M8528-P2. Two IPMC strips are joined at the ends and powered at one end to form the actuator. This configuration is used since it is mechanically more stable compared to a single IPMC.

In this paper, three applications for the novel PVEH are demonstrated with experimental results, where commercially available devices have been used. For all three applications, the PVEH as described above is used.

### 2.1. Application 1

Strain gauges are small and sensitive devices. The resistance of a strain gauge changes when a force is applied to it. Strain gauges are used to measure changes in pressure, force, or tension. They are widely used in monitoring the strain experienced by structures such as bridges, buildings, aircraft, wind turbine blades, ships, and so on. The strain values of a structure can indicate various types of failure and the life left for a safe operation. Hence, monitoring the strain experienced at critical locations of a structure is important. Stress experienced at various locations on bridges, buildings, and other concrete structures can be monitored by embedding strain gauges at these locations. In aerospace applications, strain gauges are bonded to load-bearing components such as wings and rotor blades. This is essential to ensure flight safety. In railway applications, strain gauges are bonded to railway lines to monitor the stress experienced. In automobiles, they are bonded to the wheels to measure pressure. This helps to ensure safety and timely maintenance.

It is important to ensure a reliable power source for the strain gauges. A conventional wired system is not practical in many cases where the structure is inaccessible or located in hostile conditions, hence the need for an autonomous power supply that harvests energy from the environment.

**Figure 2:**
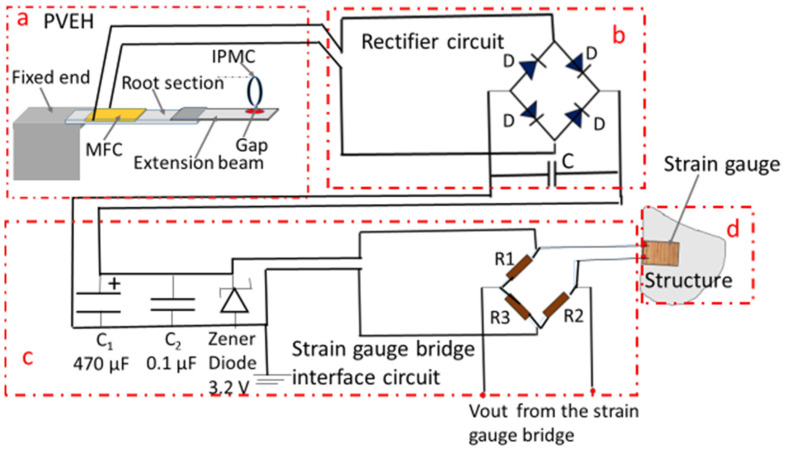
Circuit for powering a strain gauge bridge using the PVEH output. (**a**) PVEH; (**b**) Rectifier circuit; (**c**) Strain gauge bridge interface circuit; (**d**) Structure on which strain gauge is fixed.

A study to power a strain gauge using the novel tunable PVEH, which harvests power from the vibrations present in the environment, is detailed below. Figure 2 shows the circuit used for the above study. The setup has 4 sections: (a) the tunable PVEH, (b) the rectifier circuit, (c) the interface circuit for the strain gauge bridge, and (d) the strain gauge bonded to the structure.

The strain gauge can be wired in different configurations, namely quarter-bridge, half-bridge, or full-bridge. In the present study, the strain gauge is wired in a quarter-bridge configuration, where it forms one active arm of a Wheatstone bridge. In order to reduce the power consumption of the strain gauges, a high-resistance strain gauge (of the order of kΩ) and balancing resistors of high value can be used. Here, however, available strain gauges with 120 Ω resistance (Micro Measurements, Raleigh, NC, USA) and balancing resistors of 120 Ω for R_2_ and 1200 Ω for R_1_ and R_3_ are used.

**Figure 3:**
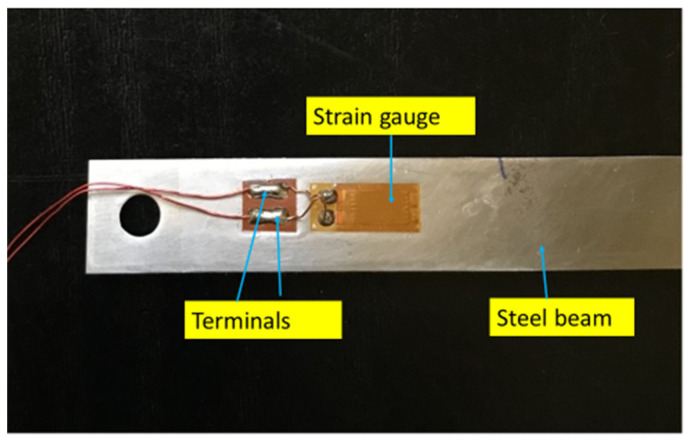
Steel beam with a strain gauge bonded to it.

**Figure 4:**
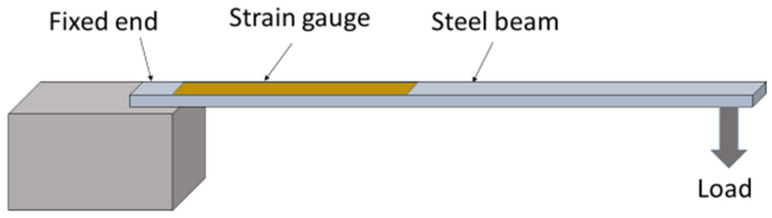
Experimental setup of the strain-gauged beam.

In the real scenario, structures will be subjected to strain at different locations when they undergo loading during their operational cycles. The corresponding strain on the structures has to be monitored in order to ensure that no catastrophic failure happens. For this, a PVEH can be deployed on the structure so that the vibrations can generate power output, which can be used to power the strain gauges on the structure. In order to simulate a similar condition in the laboratory, a steel beam of length 11.5 cm and width 1 cm with a strain gauge (Micro Measurements) bonded to it using the prescribed bonding glue was used for the experiment. The photograph of the beam is given in Figure 3. The beam was fixed at one end as in Figure 4 and loaded at the free end in steps to generate a bending of the beam. This bending generates a strain on the surface to physically extend the strain gauge. This extension of the strain gauge changes its resistance value. When this strain gauge is connected to one of the arms of a Wheatstone bridge, the output of the circuit gives a voltage corresponding to the changed strain value. The tunable PVEH was subjected to vibrations and operated at the resonant frequency. The output of the strain gauge on the Wheatstone bridge was monitored.

Using this novel PVEH configuration, it is observed that the output of the bridge is maintained even when the ambient frequency drifts slightly away from the resonant frequency due to the nonlinear response of the harvester. Since the power generated is continuously stored, this acts like a stable source of power.

### 2.2. Application 2

**Figure 5:**
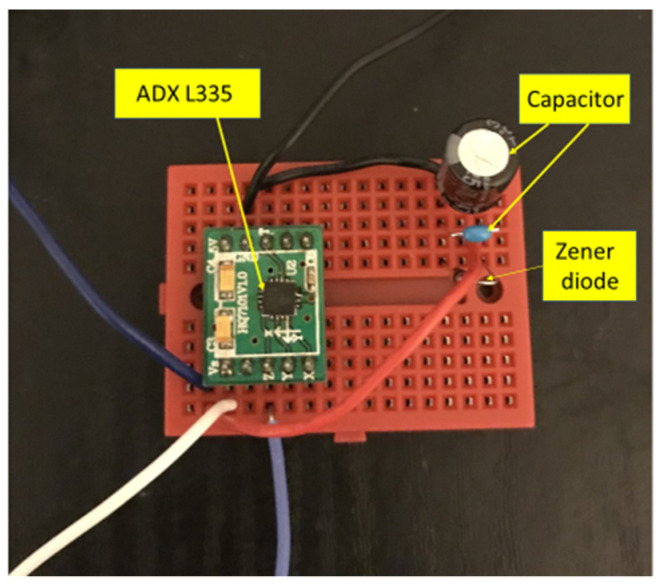
ADX L335 MEMS accelerometer and circuit.

As a second application, a tri-axial MEMS accelerometer was selected for the experiments. ADX L335 is a tri-axial accelerometer from Analog Devices that works at 2 to 3.3 V DC and draws a current of 300 µA. Figure 5 depicts the ADX L335 accelerometer and circuit used. A tri-axial accelerometer is used for motion detection in various applications of surveillance and security. In most such applications, the accelerometer will have to be deployed in inaccessible locations, which makes wired power using a conventional power supply impossible. Hence, such a scenario calls for an autonomous power source for the sensor.

**Figure 6:**
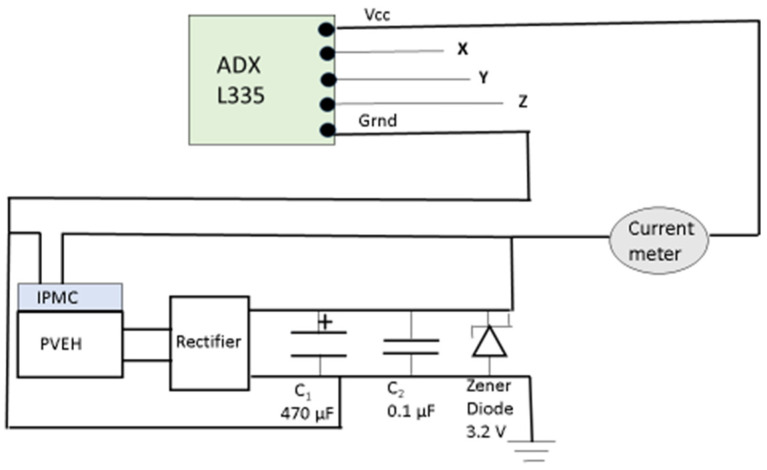
Circuit for powering the MEMS accelerometer and IPMC using PVEH output.

Figure 6 shows the connection diagram of the interface circuit with the PVEH output. The PVEH output was connected to a full-wave rectifier, and the output of the rectifier was connected across an electrolytic capacitor C_1_ of 470 µF, another capacitor C_2_ of 0.1 µF, and a Zener diode of 3.2 V. C_1_ was used to store the charge developed, and C_2_ was used to smooth the signal. The Zener diode was used to regulate the voltage given to the accelerometer so that it did not exceed the rated 3.3 V.

The analog output of the ADX L335 can be monitored in the X, Y, and Z directions when there is a motion in the X, Y, and Z directions, respectively. The output of the Zener diode was also connected to the IPMC. The PVEH was operated by an electrodynamic exciter, which was driven by a function generator and a power amplifier. It was found that the power generated by the PVEH could power both the accelerometer and the IPMC.

### 2.3. Application 3

In a third application, the PVEH was used to power a Bluetooth PASCO accelerometer along with its transmitter. The vibration signature of a structure is an important input for the diagnosis and prognosis of structural health. Many structures that vibrate are not static, such as rotating gears and rotor blades. In such structures, monitoring the vibration signature over time is a challenge because the wired powering of sensors is not feasible. Here, an autonomous power source such as a PVEH has great applications. In this application, the sensor output has to be transmitted to a remote station. Hence, both the sensor and the transmitter have to be powered using the PVEH.

In this case, the charge generated from the PVEH was stored in a supercapacitor and interfaced with the Bluetooth device.

**Figure 7:**
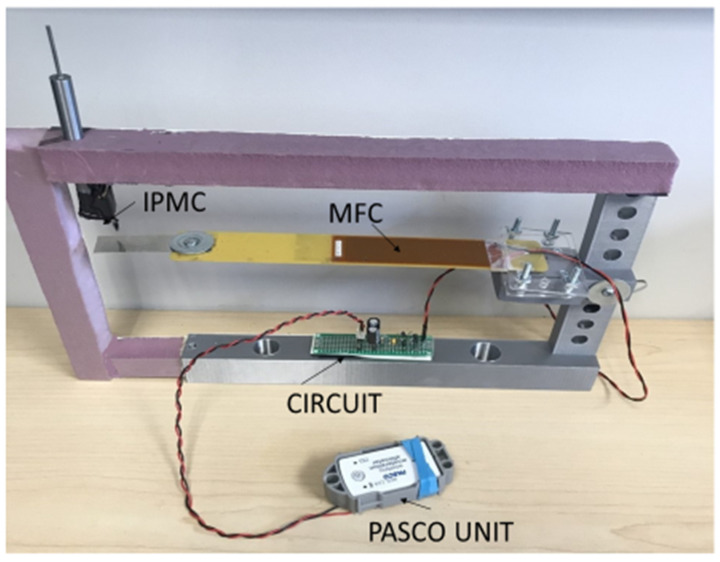
Setup of the PVEH powering the PASCO Bluetooth device.

**Figure 8:**
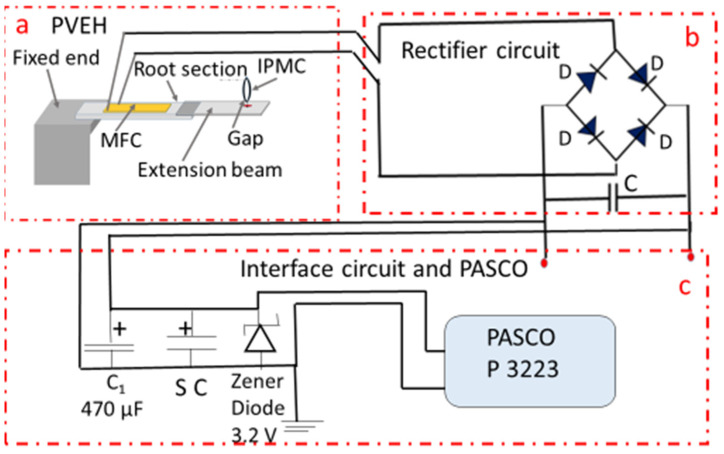
Circuit of the setup for powering the PASCO accelerometer/transmitter unit. (**a**) PVEH; (**b**) Rectifier circuit; (**c**) Interface circuit and PASCO unit.

PS 3223 accelerometer/transmitter was used for this application. This is a wireless 3-axis acceleration/altimeter Bluetooth unit from PASCO that can transmit data to a remote receiver with the corresponding software. Figure 7 shows the setup for powering the PASCO unit. Figure 8 gives the circuit diagram of the experiment.

In the circuit, SC is the supercapacitor of 1F, rated for 5.5 V (DigiKey DGH105Q5RS, Thief River Falls, MN, USA). Initial charging took 3 h 20 min to charge the supercapacitor to 3 V.

## 3. Results and Discussion

**Figure 9:**
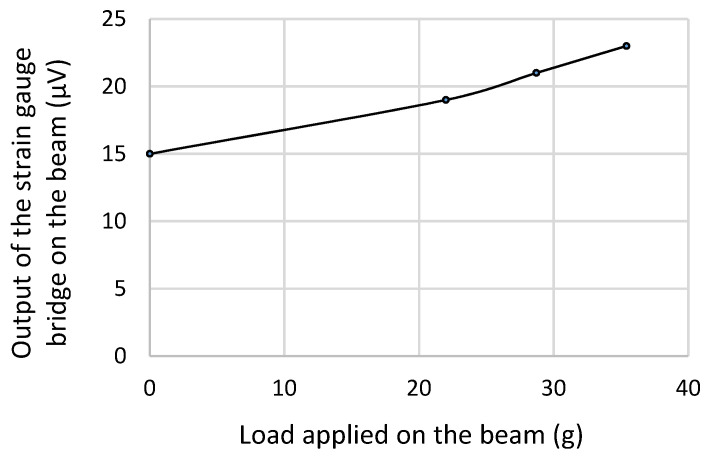
Response of the strain gauge bridge powered by the harvester.

In the first application, the strain gauge bridge circuit gave an output that increased with an increase in the load applied (between 20 g and 35 g) to the strain gauged beam. Figure 9 depicts this response, and it can be seen that it is linear. The stable output of the strain gauge at different loading conditions indicates the presence of a constant power source that is enabled by the PVEH with the interface circuit.

The stable output across the storage capacitor and the Zener diode that is connected to the Wheatstone bridge makes it possible to obtain a consistent output from the strain gauge, which is important in the measurement.

The second application of powering the accelerometer proves that the harvester could generate output sufficient to power the accelerometer along with the IPMC. The PVEH was in resonance at 8.8 Hz and generated 2.03 V across the Zener diode. The total current drawn was 250 µA. The accelerometer gave an output voltage varying from 1.5 V to 2.7 V depending on the position as it moved along the X, Y, or Z directions. The accelerometer and the IPMC could be powered by the harvested power.

In the third application, the CR 2032 3 V cell of the PASCO unit was replaced with the output from the harvester. By doing so, the PASCO unit could be successfully powered to transmit signals. The transmitted signals were acquired remotely using the data acquisition software SPARKvue. When the PVEH was subjected to vibration, it kept charging the supercapacitor. The Zener diode of the selected rating enables the required constant power supply to the sensor.

Other low-power devices widely used can also be powered by this novel device. The proposed PVEH can be designed in a MEMS configuration since the basic cantilever configuration and the IPMC actuator can be miniaturized. Further, IPMC can be configured in multiple ways. IPMC in stacked configuration can have a higher blocking force. A tri-axial accelerometer that takes just 23 µW can also be integrated for sensing the ambient frequency to enable autonomous functioning. A proposed design to implement the same is detailed below:

### Future Work: Proposed Microelectromechanical System (MEMS) Design of the Tunable Piezoelectric Vibration Energy Harvester (PVEH)

**Figure 10:**
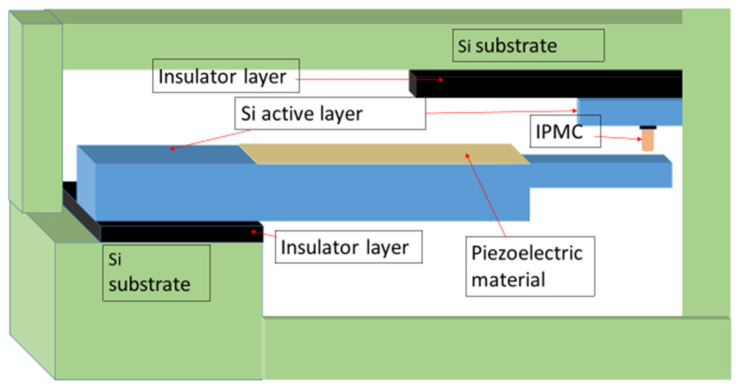
MEMS scheme for the novel design of tunable PVEH based on the SOI process.

A scheme for fabricating the tunable PVEH in MEMS configuration, considering the process details suitable for the PVEH cantilever and the IPMC (ionic polymer–metal composite) actuator, is given in Figure 10. The salient features of IPMC make it well suited for miniaturization.

The silicon-on-insulator (SOI) process is proposed here, considering the various advantages [28]. Most importantly, in the SOI-based MEMS process, it is possible to combine bulk and micromachining methods as required. The insulating layer is typically silicon oxide, and the top active silicon layer will be crystalline silicon. In Figure 8, the active layer is the cantilever beam with the required step configuration, on which the piezoelectric material is deposited. The substrate silicon layer will be of the order of a few hundreds of microns. The insulating layer will have a thickness of one micron or less, and the device layer (active layer) will have a thickness of tens of microns. The interdigitated electrode pattern that will be used in the device will enable maximum power transfer.

The PVEH can be integrated with the IPMC, which functions as a stopper. The literature clearly indicates that IPMC is highly suitable for MEMS-scale fabrication [29,30,31,32]. The above-mentioned Si-based process also works well for IPMC. It can be formed at the microscale and with different orientations of actuation. IPMC MEMS can be easily integrated with other electronics, which will be useful in this present device scheme. Distributed micro-actuators can also be fabricated using IPMC.

The whole device can be batch-produced in large numbers and at a lower cost. Considering the wide applications of the tunable PVEH across the fields of structural health monitoring and biological applications of powering implantable devices [32,33], this MEMS version of the device will have many applications.

## 4. Conclusions

1. The novel tunable PVEH can continuously provide power to low-power devices such as sensors and transmitters.
2. The device generates power at the adjacent frequencies to the basic resonant frequency also, due to the stopper action of IPMC.
3. The proposed PVEH, fabricated in MEMS form, can be integrated with other low-power sensors, transmitters, and microcontrollers as required for different applications.
4. The MEMS version of the novel device can be easily integrated with sensor arrays.

## 5. Patents

Dr. Rishi Gupta and Sreekumari Raghavan have a US patent pending—application No. 20210159816.

## Data Availability

All data is provided in the manuscript itself.
